# The challenges in data integration – heterogeneity and complexity in clinical trials and patient registries of Systemic Lupus Erythematosus

**DOI:** 10.1186/s12874-020-01057-0

**Published:** 2020-06-24

**Authors:** Helen Le Sueur, Ian N. Bruce, Nophar Geifman, on behalf of the MASTERPLANS Consortium

**Affiliations:** 1 Centre for Health Informatics, Vaughan Housue, Portsmouth St., The University of Manchester, Manchester, M13 9GB UK; 2 Arthritis Research UK Centre for Epidemiology, The University of Manchester, Manchester, UK; 3 NIHR Manchester Biomedical Research Centre, Manchester University Hospitals NHS Foundation Trust, Manchester Academic Health Science Centre, Manchester, UK; 4 The Manchester Molecular Pathology Innovation Centre, The University of Manchester, Manchester, UK

**Keywords:** Data integration, Data harmonisation, Clinical trials, Lupus, Pooled analysis

## Abstract

**Background:**

Individual clinical trials and cohort studies are a useful source of data, often under-utilised once a study has ended. Pooling data from multiple sources could increase sample sizes and allow for further investigation of treatment effects; even if the original trial did not meet its primary goals. Through the MASTERPLANS (MAximizing Sle ThERapeutic PotentiaL by Application of Novel and Stratified approaches) national consortium, focused on Systemic Lupus Erythematosus (SLE), we have gained valuable real-world experiences in aligning, harmonising and combining data from multiple studies and trials, specifically where standards for data capture, representation and documentation, were not used or were unavailable. This was not without challenges arising both from the inherent complexity of the disease and from differences in the way data were captured and represented across different studies.

**Main body:**

Data were, unavoidably, aligned by hand, matching up equivalent or similar patient variables across the different studies. Heterogeneity-related issues were tackled and data were cleaned, organised and combined, resulting in a single large dataset ready for analysis. Overcoming these hurdles, often seen in large-scale data harmonization and integration endeavours of legacy datasets, was made possible within a realistic timescale and limited resource by focusing on specific research questions driven by the aims of MASTERPLANS. Here we describe our experiences tackling the complexities in the integration of large, diverse datasets, and the lessons learned.

**Conclusions:**

Harmonising data across studies can be complex, and time and resource consuming. The work carried out here highlights the importance of using standards for data capture, recording, and representation, to facilitate both the integration of large datasets and comparison between studies. Where standards are not implemented at the source harmonisation is still possible by taking a flexible approach, with systematic preparation, and a focus on specific research questions.

## Background

SLE is a chronic autoimmune disease, affecting different body organs, which presents with a range of symptoms and clinical manifestations. Whilst several therapies with differing modes of action are currently in use, individual response varies and overall, response rates are only 40–60% to any given treatment. MASTERPLANS, a national consortium, endeavours to improve on the current trial and error approach employed in tailoring of care, by taking a precision medicine approach; improving care for patients by identifying groups who respond well to particular therapies, and the factors that may predict response to therapy.

MASTERPLANS has gained access to a wealth of data, from a range of past clinical trials and patient cohort studies; several of which have failed to demonstrate efficacy of the drug investigated, when compared to standard of care or placebo [1, 2]. Despite the lack of detected treatment effects, data from these studies could still provide a valuable source of information. Pooling of patient-level data can increase sample sizes and provide new opportunities for analysis, while various statistical approaches can be employed to handle potential study-specific effects. Framing new research questions around groups of patients combined across studies could enable both better understanding of treatment effects, and of patient characteristics that differ between these groups.

## Main text

### Accomplishing data harmonisation

Legacy data can be extremely useful, however harmonising and combining large amounts of data from disparate datasets is not always straightforward [3–5]. Data integration would be made easier if data standards were consistently applied at the source. The Clinical Data Interchange Standards Consortium provides standards for data collection, capture, and representation, to improve accessibility, interoperability, and reusability of data for better clarity in clinical research [6]. Several different frameworks and guidelines have also been developed to assist with tackling issues related to data integration across different studies [7–11]. Other initiatives have focused on developing therapeutic-area specific data standards that could better enable data integration; such as in Polycystic Kidney Disease [12], as well as for over 30 other disease areas [6]. While using these is clearly the way forward for improving integrative research that relies on several data resources, many datasets, particularly legacy data, have not followed such guidelines or applied standards. Projects that want to integrate and use these data are then faced with the burden of aligning to standards; this is not always planned for in advance or resources may be limited. Prospective alignment of datasets, without the availability of standards, is labour-intensive and often impossible to achieve perfectly. In such cases, little practical guidance, taken from real case experiences, is available to those undertaking this process, with few, if any details given where similar work has been carried out. To begin with, understanding the content of large datasets and then aligning variables to a common data model, based on similarity is extremely time-consuming. This process of alignment is undertaken manually due to nuanced complexities that require human interpretation and knowledge with regards to differences in the way data is captured and identified within the different studies being integrated. The successful process requires good documentation and comprehensive data dictionaries, explaining the content of table fields and outlining calculations that were made. Furthermore, the complexity of the integration problem only becomes apparent once alignment has taken place. In some medical fields, for example: cancer, outcome measures are well-defined clinically and recorded in a standard universal way. However this is not the case in many more complex and multi-faceted diseases, such as lupus. Differences in the response measures taken across studies can affect how response (e.g. remission and low disease activity) can be defined in the integrated set; responders may be defined using a response measure in one study that was not collected in another study. Projects such as MASTERPLANS must therefore tackle this additional level of complexity that is inherent in the study of conditions such as SLE.

We propose that prospective alignments of unstandardised data is achievable with limited resource when specific research questions are used to direct which data are to be integrated across studies.

Here we describe our own experience applying this approach in the integration of data from four lupus studies. These studies included i) The Aspreva Lupus Management Study (ALMS) study, a prospective randomized trial aimed at assessing the efficacy and safety of long term mycophenolate mofetil (MMF) compared to azathioprine and cyclophosphamide in patients with SLE [13]; ii) The LUNAR randomized, placebo-controlled trail evaluating the efficacy and safety of rituximab [2]; iii) EXPLORER, a second randomized, placebo-controlled of rituximab with background treatment distributed among azathioprine, mycophenolate mofetil and methotrexate [1]; and iv) British Isles Lupus Assessment Group Biologics Register (BILAG BR) a national patient registry looking at the safety and effectiveness of biologic and bio-similar treatment for SLE [14].

In order to carry out analyses on groups of patients across more than one study, relevant and overlapping variables were extracted, harmonised and combined into common tables, taking a common data model approach. To simplify and make this laborious process more efficient, only variables likely to be relevant to answering specific, predefined research questions regarding treatment effects and patient characteristics were extracted, so that the amount of data to be handled was kept to a minimum. Variable choice was driven by research questions related to the MASTERPLANS project plan.

Existing documentation detailing the data tables and available variables was used to identify relevant and similar variables from each study and to ascertain which tables from each dataset should be extracted. Unfortunately, the different studies did not use similar data coding standards, making data extraction more complex. These raw data tables, containing variables of interest, underwent initial cleaning, removal of duplicate rows and superfluous variables. Each of these files were read into Matlab and organised to reflect the content of the data and structure of the tables. Separate structures were thereby created for demographics, visit variables, study drug variables, use of steroids, response variables and lab chemistry variables. To integrate across studies, the variables that contained the same or similar information from each study were identified manually and assigned to the same column in merged data-frame. Each variable was then extracted from the raw data tables for each study and assigned to the correct column, placing the data for each subsequent study sequentially in the same column. These data were then harmonised and cleaned further.

**Fig. 1:**
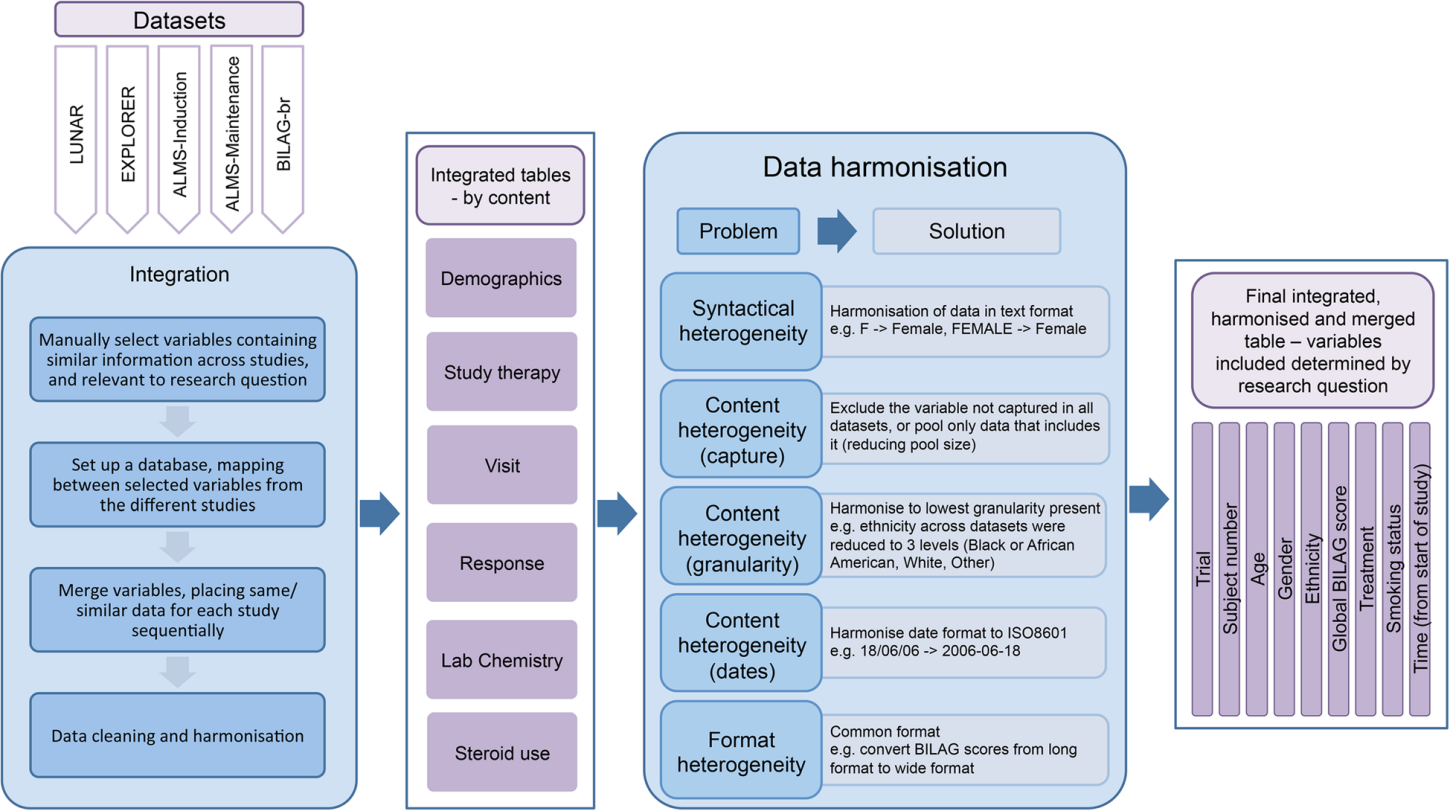
Summary of the data flow from individual datasets to final integrated, harmonised table, including examples of the changes made to the data

Lengthy processes were carried out to integrate and harmonise the data, so that each trial contained comparable information that could be analysed together (Fig. 1). The general procedure outlined here, can be used as a more generalised guide for how to carry out similar work in the future. We encountered and tackled several issues around data heterogeneity. These could be categorised into types: (i) syntactical heterogeneity, where the meaning of the data captured is the same across sources, but the words used to capture the information are different between different datasets; (ii) content heterogeneity (capture), where a whole variable is captured in one study and not in another; (iii) content heterogeneity (granularity); where, for example in ethnicity, some datasets include more categories and subsets than others, or where visit time is captured as sequential visit numbers (i.e. visit 1, visit 2 etc) in one study but as time from baseline (in days, weeks, or months) in another; (iv) format heterogeneity (variable) where the same information, for example dates, is captured in different formats; and (v) format heterogeneity (dataset/table), where, for example, a variable is captured in a single row per patient across many columns in one dataset (wide format), but in many rows per patient and in one column in another dataset (long format). In addition, further steps were taken to keep only those patients who had complete and accurate information. For example, rows with missing or mistyped dates were removed, patients were excluded if they were missing key information (e.g. treatment, follow up visits, British Isles Lupus Activity Group - BILAG response measures) and patients were excluded if they were not on their first biologic treatment. One limitation of this approach is the inevitable loss of information either due to differences in granularity or data capture; another is that the final patient group may suffer from selection biases. For example, patients on a second biologic may have more severe and/or more refractory disease and so excluding this group changes the attributes of the patients who are being analysed. Furthermore, excluding patients and rows of data based on missingness reduces the sample sizes and again, introduces potential biases.

## Conclusions

Our integration and harmonisation across SLE studies took a considerable length of time and is very specific to the data provided to MASTERPLANS. A more flexible, automated approach would significantly benefit future similar projects. Many of the steps carried out to generate the harmonised data resource required manual evaluation and examination of the data by an experienced data analyst; this is a tremendous waste of resource that is not uncommon in such projects. The work carried out here highlights the importance of standardisation, particularly regarding validated measures of disease response across both clinical trials and patient registries, at the clinical level. Application of standards for recording data at the source, measures that need including, and the format of variables, will facilitate both the integration of large datasets and comparison between studies. Significant efforts are being made to implement standards, such as the Fast Healthcare Interoperability Resources (FHIR) [8], across healthcare and medical research; however many data resources, especially legacy datasets, remain unstandardised. Where standards are not implemented at the source, reality dictates having to make a compromise in setting the approach; we argue that this can be made easier when specific research questions are used to direct which data are to be integrated across studies.

Similar integration work would benefit from the input of a data management specialist at the earliest stages in the conception of a project or trial. This would also allow for standardisation of the resulting integrated dataset, benefiting future investigations.

Despite the limitations, this work provides a useful, generalised procedure to address the known complexities in the integration of large datasets. Three key approaches were vital to the success of this work. Systematic preparation regarding the data alignment before starting to integrate was essential. A flexible approach, enabling the addition of new variables and new datasets, meant that the resulting output could be updated easily to answer new research questions if required. Finally, a focus on specific, well-defined, research questions meant that the dataset size remained manageable and was tailored by design for its intended use.

## Data Availability

To access data available to the MASTERPLANS project (http://www.lupusmasterplans.org/home.html), please contact Thomas Schindler at Roche (thomas.schindler@roche.com) for LUNAR and EXPLORER, Neil Solomons at Vifor/Aurinia (nsolomons@auriniapharma.com) for ALMS, and Emily Sutton (Emily.Sutton@manchester.ac.uk) for BILAG-BR. To access the documentation detailing the harmonisation process, please contact Dr. Patrick Doherty (Patrick.A.Doherty@manchester.ac.uk).

## Abbreviations

SLE: Systemic lupus erythematosus
*MASTERPLANS*: MAximizing Sle ThERapeutic PotentiaL by application of novel and stratified approaches
